# Science hackathons for developing interdisciplinary research and collaborations

**DOI:** 10.7554/eLife.09944

**Published:** 2015-07-23

**Authors:** Derek Groen, Ben Calderhead

**Affiliations:** Centre for Computational Science, University College London, London, United Kingdomd.groen@ucl.ac.uk; Faculty of Natural Sciences and Department of Mathematics, Imperial College London, London, United Kingdomb.calderhead@imperial.ac.uk

**Keywords:** cutting edge, careers in science, interdisciplinary research, collaboration, early career researcher, hackathon

## Abstract

Science hackathons can help academics, particularly those in the early stage of their careers, to build collaborations and write research proposals.

## Introduction

Early career researchers reside in a high-pressure environment. In addition to publishing high-quality papers, applicants for widely-coveted fellowship and lectureship positions have to show clear indications of independent research, collaborations and leadership skills. Demonstrating this is not an easy task, particularly since most early career researchers are paid to work on supervised research projects, which hinders them from becoming truly independent and assuming leadership roles.

For the purpose of aiding this transition between postdoc and fully independent researcher, we introduce the concept of a science hackathon. These events offer early career researchers the opportunity to be exposed to new scientific research questions, to explore how their own ideas might fit into the larger research landscape, and to develop their own research agenda. We discuss the merits and challenges, based on our own experiences of organizing a series of science hackathons. Following on from the success of the first event in September 2014 (which we summarize in Box 1), we are currently organizing two additional science hackathons, which will take place in the second half of 2015.

Box 1. **The First ‘2020 Science’ Hackathon.**

We held the first science hackathon in Flore, Northamptonshire, with funding support from the Software Sustainability Institute and the ‘2020 Science’ programme, both funded in the UK by EPSRC. We ran five projects with 22 participants in total, which led to six paper drafts and two software packages. For example, we ran a protocol paper project on Bayesian inference of animal receptor models and another project on a comparison of code development approaches and techniques in academia. Overall, the event was a great success with highly positive feedback from the participants. Both funding sources were keen to support similar events in the future. At this time, we have funding for two more science hackathons, and are planning to organize these in the fall of 2015. For more details of our past events, please refer to the following blog post: http://www.software.ac.uk/blog/2014-11-13-first-science-paper-hackathon-how-did-it-go.

## What is a science hackathon?

A science hackathon is an event where groups of early career researchers are able to work together on new interdisciplinary projects for a concentrated period of time, usually over the course of around three days. The aim of a hackathon is to bring together researchers with complementary skills and knowledge; they then work together to create an initial scientific write-up and research plan, which can provide the momentum needed to get a new collaboration off the ground. The write-up can subsequently form the basis for a collaborative funding proposal and, eventually, a research paper. This offers the researchers the opportunity to establish new and completely independent collaborations with minimal intrusion into their normal duties, and in the process create a basis for long-lasting interdisciplinary research projects.

**Figure 1.**
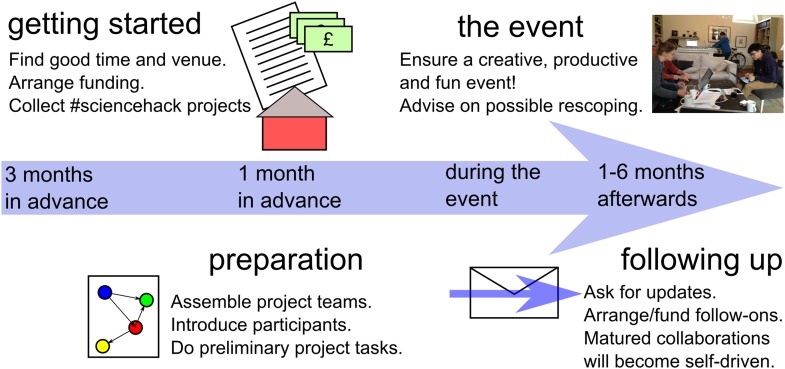
Overview of the main tasks that contribute to a successful science hackathon.

The hackathon format exists in many forms and the computer programming hackathon has found particularly widespread adoption in technology communities. The science hackathon is different in a number of important ways. Primarily, science hackathons do not feature the competitive element commonly found in programming hackathons, as we believe a non-competitive atmosphere between projects helps stimulate creativity and allows for more open discussions between groups. In addition, the incentives for a science hackathon are arguably more long-term than those for a programming hackathon and include, for example, preparing a paper, writing a proposal for funding, and/or making steps towards becoming a more independent researcher. In Figure 1, we provide an overview of the steps needed to create, run, manage and consolidate science hackathon projects.

Science hackathons do not feature the competitive element commonly found in programming hackathons, as we believe a non-competitive atmosphere helps stimulate creativity and allows for more open discussions between groups.

## Engineering a successful science hackathon project

There are several aspects that we believe help define a strong project. Firstly, the organization of individual projects is important. Before the event, participants have the opportunity to propose suitable projects. A scientific organizing committee must then try to select a number of these and match all other participants and their skills with suitable project partners. The early career researcher proposing the project will usually take the overall lead on their idea. However, this does not preclude other team members taking the lead on individual aspects of the project. This is especially feasible with interdisciplinary work, whereby one person may lead on the statistical methodology, another may lead on the computational aspects, while another still may assume ownership of the experimental research. This mix of complementary scientific perspectives allows for more individuals to develop their own leadership skills and take ownership of different ideas within a single research project.

In order to increase the impact of a science hackathon, we believe that it is vital to choose well-designed individual projects that have achievable short-term goals, yet are extendable into more developed longer-term scientific investigations. Research is by its nature a risky endeavour; we often don't know in advance what is possible until we try it, and research directions will often change as a project develops. By focusing on a small task, however, we may often gain insight into the bigger challenges that arise in the overall research. Depending on the field of research, suitable short-term aims could include: the exploratory analysis of an existing dataset; writing proof-of-concept computer code that applies existing methodology to a new problem; developing a plausible mathematical model for describing a biological system or natural phenomenon of interest; creating a skeleton draft of a paper; or writing an initial draft of a funding proposal.

Although the aim of these short, intense projects is not necessarily to create a finished research paper, having an explicit goal of building towards a publication or funding proposal from the outset helps crystallize ideas about research direction. This also provides a strong incentive for early career researchers to contribute and invest time into the collaboration long after the event. We suspect that making a project plan alone is unlikely to provide sufficient momentum; it is easier for early career researchers to justify working on projects that are already well underway, with existing computer code to build on, a basic mathematical model to extend, or a research grant outline to be developed.

## Selecting hackathon teams

In our experience, hackathon projects work very well when teams consist of researchers from diverse experimental and computational fields. At this fruitful interface, there exists the potential for the rapid cross-fertilization of ideas that have not yet been fully explored. Furthermore, groups containing people with diverse and complementary areas of expertise may facilitate research that cannot be achieved by any one individual member working alone, and may lead to combined research output that is greater than the sum of its parts.

This diversity is also important for allowing the work to be divided and worked on in parallel; during a science hackathon a team of early career researchers will work on the project simultaneously, so it is important that the overall project can be broken down into smaller building blocks that link together. For example, a couple of participants could develop a mathematical model, while other participants work on developing computer code or draft an introductory section for the write-up, outlining the possible directions for the research. The key is that each member is able to contribute their unique expertise to this interdisciplinary hackathon project.

We believe that having the right team size is also very important. ‘Regular’ hackathons most commonly have projects with three to six participants each, and we have found that this size of team also works well for science hackathons. This allows each team to contain a large enough variety of perspectives while still being small enough to allow the groups to rapidly form a consensus and make progress on the research topic.

## Practicalities of running a science hackathon event

**Figure 2.**
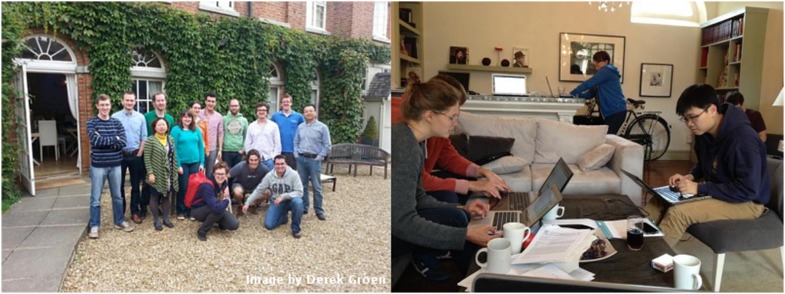
Left: Group photo of a subset of participants (since several of them were still working on their projects). Right: A project group working during the event.

Science hackathon projects are meant to be highly ambitious, and for this reason the environment in which the projects take place is absolutely crucial. In our experience, we found it helpful to find a remote venue that was conducive to free thought and concentration, implicitly nudging participants to fully commit to the project during the entire hackathon with minimal external distraction. In our case, we opted for a self-contained conference venue with a garden and usable outdoor space in a tiny town in Northamptonshire for two and a half days (see Figure 2 for several impressions of the first event and the venue), although in retrospect we feel that a slightly longer duration would have been beneficial.

Like other hackathons, science hackathons are very intensive, and as organizers there are a number of things we can do to help participants stay focused, energetic and inspired with creativity. Firstly, it really helps to have good quality food and drink (and a plentiful supply of coffee/tea) available throughout the day, and to have all the activities (sleeping, sciencehacking, eating and so on) in the same location, to avoid the overhead of travel. In our self-catering venue we organized a timetable for each of the groups to participate in cooking and cleaning for each other, which helped further develop the sense of camaraderie and teamwork.

Secondly, in order to keep the participants strongly focused, we found it helped to keep any talks/presentations to the bare minimum, since they tend to break the flow of the event. Any presentations were therefore scheduled for the beginning or end of the hackathon. In general, we found it helpful to keep the schedule light and flexible to allow the research projects to develop organically.

Finally, for enhancing creativity, we tried to choose an unconventional location with alternative facilities for relaxation (in our case there was an indoor swimming pool at the venue). We kept the event very informal and deliberately chose not to introduce any element of competition, so that everyone could focus on their own work, rather than worrying about what the other groups were doing.

The timing of the science hackathon is also very important. We tried to increase attendance by choosing a period when the weather is likely to be good, and when researchers are not likely to be giving lectures or attending other events. Intensive events like a science hackathon need a little time to gain momentum, and we found that the two and a half days we allocated for our first hackathon was probably the minimum duration to get a good momentum going.

## Keeping the momentum going

Successful science hackathon projects establish a foothold for further academic collaboration, and seldom result in a finished product themselves. Therefore, we recommend facilitating follow-ups in the weeks after the event. The simplest way to do this is by engaging with the team leaders on a monthly basis about the progress of their projects, and offering further assistance in developing their collaboration. In our case, we arranged funding for follow-up meetings, where various groups can come together and strengthen their new research agendas. As the established collaborations mature, we expect them to become more self-driven and self-funded.

The five projects we ran during the first Science Hackathon event were on the whole very successful. At time of writing, the projects have resulted in one journal paper undergoing review (on software development practices in academia), one paper which is undergoing a pre-submission inquiry (on software reproducibility of scientific results), one book chapter which is under review (on writing a numerical solver using the newly developed JULIA programming language), and one code base which has been released on GitHub and is actively being maintained (https://github.com/UCL/LatBo.jl). Finally, another one of the teams is nearing completion on a paper draft investigating a Bayesian analysis of photoreceptor models. The first science hackathon event has therefore resulted in many collaborations and interdisciplinary pieces of work between participants who would not otherwise have engaged with one another, and we look forward to discovering the new collaborations that will arise from our planned science hackathon events in the near future.

